# Prevalence and correlates of stroke among older adults in Ghana: Evidence from the Study on Global AGEing and adult health (SAGE)

**DOI:** 10.1371/journal.pone.0212623

**Published:** 2019-03-13

**Authors:** Olutobi Adekunle Sanuade, Francis Nii-Amoo Dodoo, Kwadwo Koram, Ama de-Graft Aikins

**Affiliations:** 1 Institute of Advanced Studies, University College London, London, United Kingdom; 2 Regional Institute for Population Studies, University of Ghana, Accra, Ghana; 3 Noguchi Memorial Institute for Medical Research, University of Ghana., Accra, Ghana; Monash University, AUSTRALIA

## Abstract

This study examines the prevalence and correlates of stroke among older adults in Ghana. This cross-sectional study retrieved data from Wave 1 of the World Health Organization (WHO) Survey on Global Ageing and Adult Health (SAGE) conducted between 2007 and 2008. The sample, comprising 4,279 respondents aged 50 years and above, was analysed using descriptive statistics, cross tabulations and Chi-Square tests, and a multivariable binary logistic regression. Respondents ranged in age from 50 to 114 years, with a median age of 62 years. Stroke prevalence was 2.6%, with the correlates being marital status, level of education, employment status, and living with hypertension or diabetes. The results showed that being separated/divorced, having primary and secondary education, being unemployed and living with hypertension and diabetes, significantly increased the odds of stroke prevalence in this population. The results suggest that interventions to reduce stroke prevalence and impact must be developed alongside interventions for hypertension, diabetes and sociodemographic/economic factors such as marital status, level of education, and employment status.

## Background

Stroke is the second leading cause of death and the third leading cause of disability worldwide [1]. It is estimated that 15 million people suffer from stroke every year. Out of this number, about six million people die and another five million are left permanently disabled [2]. A total of 44 million disability-adjusted life-years (DALYs) are lost to stroke every year and this is projected to increase to 61 million by 2020 [2]. The prevalence is projected to increase throughout the world because the number of persons aged 60 years and above is expected to more than double by 2050, and more than triple by 2100, increasing from 901 million in 2015 to 2.1billion in 2050 and 3.2 billion in 2100 [3].

In low- and middle-income countries (LMICs), stroke incidence is increasing, and research has shown that stroke mortality will triple in Latin America, the Middle East, and sub-Saharan Africa between 2002 and 2020 [4]. Community-based studies in sub-Saharan Africa (SSA) show that stroke is the cause of 5–10% of all deaths, and this is partly because of inadequate health systems and increasing rates of hypertension [5]. Further, the impact of stroke is projected to go up in this region as a result of urbanization, poor socio-economic status and the change in the demographic structure of the population from young to an ageing population. By 2025, it is projected that about half of SSA’s populations will be living in urban areas and the number of people who are aged 60 years and above will more than double in countries like Ghana, Cameroon, Democratic Republic of Congo, and Mozambique [6,7]. This projected demographic transition may increase stroke-induced disability in the region in the near future if serious measures are not put in place. Studies have shown that stroke is a major cause death and disability in Ghana [8]. Also, hypertension prevalence is already high in the country, with low rates of awareness, treatment and control [9–11]. This has serious implications for the burden of stroke unless urgent measures are taken to control hypertension [9]. The current situation shows that stroke is not on the list of priority health interventions outlined by Ghana's Ministry of Health and stroke burden has been under-researched and under-funded in Ghana [12].

Research showed that the risk factors of stroke include hypertension [13–16], diabetes [15–18], dyslipidemia, cardiac disease [15, 16], smoking [1, 19–22], alcohol consumption [23–25], physical inactivity [26–29], obesity, regular meat consumption, low green leafy vegetable consumption, stress, and adding salt at the table [15, 16]. In addition, sociodemographic/economic factors such as sex [6, 30–33], age [1,34], level of education [35], wealth status [36–39] have been shown to be associated with stroke. Recent evidence by the Stroke Investigative Research and Evaluation Network (SIREN) multicenter case-control study showed the dominant modifiable risk factors of stroke in Ghana and Nigeria include hypertension, dyslipidemia, regular meat consumption, waist-to-hip ratio, diabetes, low green leafy vegetable consumption, stress, adding salt at the table, cardiac disease, physical inactivity and current cigarette smoking [16]. Nevertheless, the study provided limited information on the role of sociodemographic/economic factors on stroke prevalence and the findings cannot be generalized to the entire Ghana. Since social determinants of health (SDH) are the conditions in which people are born, grow, work, live, and age, and the fundamental drivers of these conditions [40], this indicates that their effects on stroke are context-specific and may provide important information on health disparities. To date, no study has examined the prevalence and correlates of stroke at the national level in the country, despite the importance of this evidence to drafting national guidelines and developing primary and secondary intervention strategies for the illness in Ghana. This study examines the prevalence and correlates of stroke in Ghana to address this important gap.

## Data and methods

### Study design

This cross-sectional study retrieved data from Wave 1 of the World Health Organization (WHO) survey on Global Ageing and Adult Health (SAGE) conducted between 2007 and 2008. The sample comprised 4,279 respondents aged 50 years and above.

### Study area

The 2010 Population and Housing Census showed that Ghana has a population of 24,658,823, a larger proportion (51.2%) of whom are female [41]. The sex ratio is 95.2 males per 100 females. There are ten regions in the country. Of the ten regions in the country, the most populous is the Ashanti Region, made up of 19.4% of the total population; this is followed by Greater Accra Region (16.3%) with the least populated region being the Upper West Region (2.8%). With regard to the age structure, 38.3% of Ghana’s population is less than 15 years; 49.5% is 15–49 years, and; 12.2% is aged 50 years and above [41]. Further, a little over half of the population (50.9%) is living in urban areas [41]. More than 70% are Christians, 17.6% are Muslims, 5.2% are traditional worshippers and 5.3% do not have any religious affiliation. In terms of occupation, a larger proportion engages in agriculture-related activities. Specifically, 41.2% of the economically active population is skilled agricultural, forestry and fishery workers; about 21% is also engaged as service and sales workers, while 15.2% are craft and related trade workers.

Ghana is a lower-middle income country with average per capita income of GH¢5,347(about US$1,353) [42]. The health system is structured to mainly treat infectious diseases [12]. The health care system in Ghana faces a lack of investment in health system and health workforce generally. Health spending as a percentage of GDP is 5.4% in 2013 [43], higher than the WHO recommendation of 5% [43]. There are three teaching hospitals (Korle Bu in Accra, Komfo Anokye in Kumasi and Tamale Teaching hospital); nine regional hospitals; 96 government hospitals; and 1,106 health centres and clinics [44]. In terms of the distribution of health professionals, there are 4,747 medical and dental practitioners, 1,832 physician assistants, 759, anaesthetics, 24,974 nurses, 1129 pharmacist and 41 health research officers. The doctor-population ratio was 1:11929 and the nurse to population ratio was 1:971 [41, 45].

### Sampling design

Ghana SAGE Wave 1 used a stratified, multistage cluster design that was based on the design for the World Health Survey [46] and presented a nationally representative sample. The primary sampling units were stratified by administrative region (Ashanti, Brong Ahafo, Central, Eastern, Greater Accra, Northern, Upper East, Upper West, Volta, and Western) and type of locality (urban/rural). Based on this, a total of 20 strata were developed [46, 47]. From each of the strata, a total of 10–15 Enumeration Areas (EAs) were selected according to the population size. Household listings were done for each selected EA. Twenty households with persons aged 50 years and above, and four households with persons aged 18–49 years were then selected for interview [46]. All persons aged 50^+^ in ‘older’ households (households with at least one person aged 50-plus years) were invited to participate, whereas only one person was randomly selected in the ‘younger’ households (households with no person aged 50-plus years). Further, for those who were incapable of completing an interview for reasons of health or cognition, a proxy questionnaire was completed [46]. Standardized training in all aspects of the interview was provided to all interviewers. The questionnaires were translated into respective local languages, following a translation protocol, and modified to take into account the local context where needed [20]. The interview response rate was 86% [46].

### Ethics statement

Ethical clearance and permission for the SAGE study was sought and approved by the Ethics Review Committee, World Health Organization, Geneva, Switzerland and locally from Ethics Committee, University of Ghana Medical School, Accra, Ghana. Written informed consent was obtained from all participating individuals.

### Measures

#### Stroke prevalence

Stroke prevalence was based on self-report. The specific question asked during the survey was “Have you ever been told by a health professional that you have had a stroke?” Hence, the prevalence of stroke in this study was determined as the proportion of Ghanaians aged 50 years and above who had ever been told by a health professional that they have a stroke [46]. Self-reported diagnosis has been used to ascertain stroke prevalence in similar population-based studies in Africa [48], Asia [49, 50], Oceania [51, 52], Europe [53, 54], and North America [55–57]. These studies showed that self-reported diagnosis is a valid approach for estimating stroke prevalence and has high sensitivity and specificity values [51–53, 56, 57]. During the data collection, respondents were provided with the definition of stroke. Stroke was described as a sudden and severe attack to the brain, which can cause permanent or temporary paralysis (inability to move, usually one side of the body) and loss of speech [58].

#### Physical activity

Physical activity was measured as the number of days respondents spent doing moderate-intensity activities including sports, fitness, or recreational leisure activities. This was re-categorized into three: physically inactive, partially active (those engaged in physical activities less than 3 times a week), and fully active (those who engaged in physical activities 3 or more times a week.

#### Smoking and alcohol consumption

Two questions were used to categorize smoking status. These included whether the respondent had ever smoked and if they currently smoked. These categories were re-grouped into non-smokers, previous smokers and current smokers. Those who responded ‘No’ to the two questions were referred to as ‘non-smokers’ while those who responded ‘Yes’ to whether they ever smoked and ‘No’ to whether they currently smoked were categorized as ‘previous-smokers’. Those who responded ‘yes’ to both questions were categorized as ‘current smokers’. Alcohol consumption was represented by three categories: non-drinkers (those who had ‘never’ consumed alcohol), occasional drinkers (those who had taken alcohol ‘once in a while’), and; regular drinkers (those who take alcohol ‘all the time’).

#### Body mass index

BMI was categorized according to WHO criteria (34): BMI < 18.5 (underweight), BMI = 18.5–24.99 (normal weight), BMI = 25–29.99 (overweight), and BMI ≥ 30 (obese).

#### Comorbidities

The comorbidities examined in this study were diabetes and hypertension. Respondents who had been diagnosed with hypertension by a health professional, and/or had the average of three blood pressure (BP) measurements to be systolic ≥140mm Hg, and/or diastolic BP of ≥ 90mm Hg, or were on antihypertensive medications, were regarded as living with hypertension. Diabetes prevalence was measured on a self-reported diagnosis by a health professional or use of insulin or other blood sugar lowering medications.

#### Other independent variables

The other independent variables included in this study were sex, age, place of residence, marital status, level of education, wealth status, religion, employment status and ethnicity.

### Data analysis

Descriptive statistics such as frequency distributions and median were used to describe the socioeconomic and demographic characteristics of the respondents, lifestyle factors, and comorbid conditions. At the bivariate level, cross tabulations and Chi-Square tests were used to determine the variation in stroke prevalence by socioeconomic and demographic characteristics, lifestyle factors and comorbid conditions. Further, a multivariable binary logistic regression was used to examine correlates of stroke prevalence in Ghana and the alpha level for statistical significance was set at 0.05. At the multivariable analyses, the reference categories were theoretically selected based on what existing literature has shown. For instance, research shows that stroke prevalence is relatively higher among males compared to females; hence, we made the female gender the reference category in the multivariable analysis. Further, since the WHO SAGE used a stratified, multistage cluster sampling, appropriate sampling weights were applied before the analysis was done to adjust for the survey design. We also tested for multicollinearity using variance inflation factor (VIF) and tolerance; the outcomes indicate that there was no high intercorrelations among the variables (S1 Table).

## Results

### Socioeconomic and demographic characteristics of respondents

The socioeconomic and demographic characteristics of the respondents are shown in Table 1. The respondents’ ages ranged from 50 to 114 years with a median age of 62 years. More than one-third (39.3%) were between 50–59 years while 38.0% were 60–69 years and 9.9% was 80 years and above. More than half (52.3%) was female and close to 60.0% lived in the rural areas. The highest proportion was currently married (56.8%), more than one-fourth (27.9%) was widowed and the least proportion (1.2%) was never married.

**Table 1:** Percentage distribution of the respondents by socio-demographic characteristics.

Characteristics	Number (n = 4279)	Percentage
**Sex**		
Male	2043	47.7
Female	2236	52.3
**Age Group**		
50–59	1682	39.3
60–69	1197	28.0
70–79	975	22.8
80+	425	9.9
**Place of residence**		
Rural	2531	59.2
Urban	1748	40.8
**Marital status**		
Never married	50	1.2
Currently married	2431	56.8
Separated/divorced	606	14.1
Widowed	1192	27.9
**Level of education**		
No education	2362	55.2
Primary	891	20.8
Secondary	173	4.1
Higher	853	19.9
**Wealth status**		
Poorest	852	19.9
Poorer	842	19.8
Middle	854	20.0
Richer	868	20.2
Richest	863	20.1
**Religion**		
No religion	214	5.0
Christianity	2939	68.7
Islam	673	15.7
Other	453	10.6
**Employment status**		
Unemployed	1325	31.0
Employed	2954	69.0
**Ethnicity** Akan	2052	48.8
Ewe	289	6.9
Ga Adangbe	436	10.4
Gruma	215	5.1
Grusi	42	1.0
Guan	65	1.5
Mande Busanga	63	1.5
Mole Dagbani	105	2.5
Others	939	22.3

Source: Computed from SAGE survey data, 2008

Further, more than half (55.0%) had no education and about one-fifth (19.9%) had tertiary education. With regard to wealth status, almost equal proportions were in the middle, richer and richest categories (20.0%, 20.2% and 20.1% respectively). More than six out of ten older adults (68.7%) were Christians, about 16.0% were Muslims and 5.0% had no religion. In terms of the employment status, close to 70% was employed. The largest proportion (48.8%) was Akan, followed by Ga Adangbe (10.4%), and the least proportion (1.0%) was Grusi.

### Lifestyle factors and comorbidities

The results showed that stroke prevalence was 2.6% (Table 2). While almost three out of four (74.2%) had never smoked, 13.3% and 12.5% were previous and current smokers, respectively. More than 40% had never consumed alcohol, while 28.5% were occasional consumers and 30.1% were regular consumers of alcohol. While a large proportion of the sample was not physically active (86.5%), 5.2% were partially active while 8.3% were fully active. With regard to the BMI status, more than half of the respondents (51.8%) had normal BMI; close to one-fifth (19.6%) were underweight and 28.6% were overweight or obese. More than half of the respondents (57.8%) were living with hypertension, and 4.2% were living with diabetes.

**Table 2:** Percentage distribution of respondents by lifestyle factors and comorbidities.

Lifestyle factors and comorbidities	Number (n = 4279)	Percentage
**Smoking Status**		
Non-smoker	3166	74.2
Current smokers	533	12.5
Previous smokers	570	13.3
**Alcohol consumption status**		
Non-consumer	1773	41.4
Occasional consumers	1221	28.5
Regular consumers	1285	30.1
**Physical Activity**		
Not physically active	3702	86.5
Partially active	224	5.2
Fully active	353	8.3
**BMI Status**		
Underweight	816	19.6
Normal	2157	51.8
Overweight	786	18.9
Obese	407	9.7
**Stroke**		
No	4169	97.4
Yes	110	2.6
**Hypertension**		** **
No	1804	42.2
Yes	2475	57.8
**Diabetes**		** **
No	4099	95.8
Yes	180	4.2

Source: Computed from SAGE survey data, 2008

### Univariate analysis

#### Variation in stroke prevalence by sociodemographic/socioeconomic characteristics

Table 3 shows the variation in stroke prevalence by the socioeconomic and demographic characteristics of respondents. The table shows that stroke prevalence significantly varied by age, place of residence, marital status, level of education, wealth status, employment status, and ethnicity. Specifically, stroke prevalence was higher among the older age groups. The prevalence of stroke among those who were 50–59 years, 60–69 years and 70–79 years were 1.5%, 2.8%, and 4.2%, respectively. Stroke prevalence was higher among urban dwellers compared to their rural counterparts (3.8% and 1.7%, respectively).

**Table 3:** Association between socioeconomic and demographic characteristics and stroke prevalence.

Characteristics	Stroke		Chi-Square	df
	No	Yes		
**Sex**				
Male	97.6	2.4	0.508	1
Female	97.2	2.8		
**Age Group**				
50–59	96.5	1.5	17.793***	3
60–69	97.2	2.8		
70–79	95.8	4.2		
80+	97.6	2.4		
**Place of residence**				
Rural	98.3	1.7	18.989***	1
Urban	96.2	3.8		
**Marital status**				
Never married	96.0	4.0	12.279**	3
Currently married	97.9	2.1		
Separated/divorced	95.4	4.6		
Widowed	97.4	2.6		
**Level of education**				
No education	98.3	1.7	21.581***	3
Primary	96.1	3.9		
Secondary	94.0	6.0		
Higher	97.0	3.0		
**Wealth status**				
Poorest	98.6	1.4	9.609*	4
Poorer	97.5	2.5		
Middle	97.1	2.9		
Richer	97.7	2.3		
Richest	96.3	3.7		
**Employment status**				
Unemployed	93.9	6.1	93.535***	1
Employed	99.0	1.0		
**Ethnicity**				
Akan	96.8	3.2	18.508*	8
Ewe	96.1	3.9		
Ga- Adangbe	96.4	3.6		
Gruma	98.4	1.6		
Grusi	100.0	0.0		
Guan	98.4	1.6		
Mande Busanga	96.8	3.2		
Mole Dagbani	97.1	2.9		
Others	99.0	1.0		

Source: Computed from SAGE survey data, 2008

*p <0.05

**p < 0.01

***p < 0.00; df- degrees of freedom

In addition, stroke prevalence was highest among respondents who were separated/divorced and lowest among those who were currently married (4.6% and 2.1% respectively). With regard to education, stroke prevalence was higher among those with higher level of education. Stroke prevalence was highest among those in the richest wealth quintile (3.7%) and lowest among those in the poorest wealth quintile (1.4%). Concerning variation in stroke prevalence by employment status, the results showed that stroke prevalence was significantly higher among those who were unemployed (6.1%) compared to those who were employed (1.0%). Among the ethnic groups, stroke prevalence was highest among the Ewe (3.9%) and none of the respondents who were Grusi reported any stroke prevalence.

### Variation in stroke prevalence by lifestyle factors and comorbidities

None of the lifestyle factors was significantly associated with stroke (Table 4). Nevertheless, stroke prevalence was highest among those who were previous smokers, occasional drinkers, partially or fully active, and those who were overweight/obese. Further, hypertension and diabetes were significantly associated with stroke. While 3.8% of those living with hypertension had stroke, 10.4% of those living with diabetes had stroke.

**Table 4:** Association between lifestyle factors, comorbidities and stroke prevalence.

Lifestyle factors and comorbidities	Stroke		Chi-Square	df
	No	Yes		
**Smoking Status**				
Non-smoker	97.4	2.6	0.214	2
Current smokers	98.3	1.7		
Previous smokers	96.6	3.4		
**Alcohol consumption status**				
Non-drinkers	97.5	2.5	3.245	2
Occasional drinkers	96.8	3.2		
Regular drinkers	97.9	2.1		
**Physical Activity**				
Not physically active	97.5	2.5	0.567	2
Partially active	96.8	3.2		
Fully active	96.8	3.2		
**BMI Status**				
Normal	98.6	1.4	0.167	3
Underweight	98.0	2.0		
Overweight	97.1	2.9		
Obese	97.2	2.8		
**Hypertension**				
No	99.1	0.9	35.962***	1
Yes	96.2	3.8		
**Diabetes**				
No	97.8	2.2	53.583***	1
Yes	89.6	10.4		

Source: Computed from SAGE survey data, 2008

*p <0.05

**p < 0.01

***p < 0.001; df- degrees of freedom

### Correlates of stroke

This section reports the results of the multivariable analysis. Table 5 shows that being separated/divorced, having primary and secondary education, being unemployed and living with hypertension and diabetes, were the correlates of stroke in Ghana. The results showed that the odds of stroke prevalence were 1.5 times higher among those who were separated/divorced compared to those who were currently married. Those with primary and secondary education had higher odds of stroke prevalence compared to those with no education (1.1 times and 2.4 times, respectively). Further, those who were unemployed were 2.8 times more likely to be living with stroke compared to those who were employed. Respondents living with hypertension were 2.0 times more likely to be living with stroke; those living with diabetes were likewise 3.0 times more likely to be living with stroke, compared to their respective counterparts.

**Table 5:** Correlates of stroke.

Characteristics	Odds Ratio	s.e	95% CI
**Sex**			
Female (RC)			
Male	1.622	0.557	0.825–3.191
**Age Group**			
50–59	0.480	0.257	0.167–1.381
60–69	0.811	0.402	0.305–2.156
70–79	0.767	0.371	0.295–1.990
80+ (RC)	-	-	-
**Marital Status**			
Currently married (RC)	-	-	-
Never married	3.188	3.775	0.309–32.868
Separated/divorced	2.465*	0.877	1.222–4.968
widowed	1.181	0.444	0.563–2.478
**Place of residence**			
Rural (RC)	-	-	-
Urban	1.380	0.389	0.792–2.404
**Level of education**			
No education (RC)	-	-	-
Primary	2.083*	0.693	1.082–4.011
Secondary	3.352*	1.857	1.126–9.983
Higher	1.446	0.476	0.756–2.764
**Wealth Status**			
Poorest	1.220	0.541	0.509–2.924
Poorer	0.893	0.351	0.509–2.924
Middle	1.335	0.508	0.630–2.826
Richer	0.773	0.304	0.357–1.676
Richest (RC)	-	-	-
**Employment status**			
Employed (RC)	-	-	-
Unemployed	3.780***	1.201	2.022–7.068
**Ethnicity**			
Akan (RC)	-	-	-
Ewe	1.030	0.353	0.524–2.437
Ga- Adangbe	0.942	0.335	0.467–1.897
Gruma	0.443	0.311	0.111–1.763
Guan	0.658	0.753	0.069–6.262
Mande Busanga	0.704	0.666	0.109–4.548
Mole Dagbani	2.257	1.674	0.523–9.728
Others	0.634	0.314	0.239–1.681
**Smoking Status**			
Non-smoker (RC)	-	-	-
Current smokers	0.896	0.453	0.331–2.425
Previous smokers	1.095	0.424	0.511–2.347
**Alcohol consumption status**			
Non-drinkers (RC)	-	-	-
Occasional drinkers	1.097	0.233	0.721–1.667
Regular drinkers	0.900	0.207	0.571–1.416
**Physical Activity**			
Fully active (RC)	-	-	-
Partially active	1.315	0.900	0.342–5.064
Inactive	0.673	0.287	0.262–1.728
**BMI Status**			
Normal (RC)	-	-	-
Underweight	0.660	0.287	0.280–1.555
Overweight	1.126	0.360	0.600–2.112
Obese	0.942	0.359	0.445–1.994
**Hypertension**			
No (RC)	-	-	-
Yes	3.014***	0.815	1.769–5.134
**Diabetes**			
No (RC)	-	-	-
Yes	3.953***	1.489	1.881–8.304

Source: Computed from SAGE survey data, 2008

RC- Reference Category s.e.: standard error N = 4279 CI- Confidence Interval

*p < 0.05

**p < 0.01

***p< 0.001

## Discussion

This study examined the prevalence and correlates of stroke among Ghanaians who were 50 years and above. The findings showed that stroke prevalence was 2.6% and its correlates were being separated/divorced, having primary and secondary education, being unemployed and living with hypertension or diabetes. This study showed that the stroke prevalence rate reported in this study is lower than the ones reported in similar population in China (3.1%) [49], South Africa (4.0%) [48] and Singapore (4.1%) [50].

### Sociodemographic/socioeconomic factors and stroke prevalence

The findings showed that marital status, level of education and employment status were correlates of stroke. Our findings showed that the odds of stroke prevalence were higher among those who were separated/divorced compared to those who were married. Plausible explanation for this is that marriage is a protective factor for stroke through spousal support, early health seeking, encouragement of healthy behaviours and adherence to medication [59]. Also, this association may be a reverse causality. For instance, a qualitative study on lived experience of stroke in Ghana showed that living with stroke may result in marital separation [60], thus the higher odds of stroke prevalence among those who were separated. The odds of stroke were higher among those with higher levels of education. An explanation for this is that those with low levels of education may have experienced higher mortality since higher education has a positive association with longer life expectancy [35]. Further, the results showed that those who were unemployed had higher odds of living with stroke. One plausible explanation for this is that a large proportion of those with stroke probably may have stopped working as a result of the illness. This is likely because among those who were unemployed, the mean age at which they stopped working was 41.5 years. Further, about one-third (33.6%) of these people stopped work because of health issues or disabilities. In addition, when those who were unemployed were asked whether they were looking for work, 85.5% responded in the negative. This may suggest that they were not actively looking for work probably because of the stroke. Studies have particularly shown that stroke usually comes with physical, cognitive and behavioural disabilities that limit the sufferer’s ability to be independent or engage in productive activity [61].

On the other hand, sex, age, wealth status, religion and ethnicity were not statistically related to stroke prevalence. This is not surprising because there have been contradictory findings with respect to how these sociodemographic and socioeconomic factors are associated with stroke [6, 32, 35–37, 62]. An interesting finding that is worth explaining was the non-statistical association between age and stroke prevalence. This may be because this study only considered people who were 50 years and above, and perhaps, the risk of developing stroke is the same for all age groups after 50 years in Ghana. A review study in Ghana has shown that the risk of stroke is high in the fifth decade of life [34].

### Lifestyle factors and stroke prevalence

The results showed that none of the lifestyle factors was a correlate of stroke. An explanation for this finding may be because this study used a cross-sectional data. Even though a study in the Ashanti Region of Ghana showed that current levels of smoking are higher among older adults [63], it is possible that the effect of smoking on stroke prevalence does not differ significantly beyond age 50 in Ghana. Also, the non-statistically significant association between alcohol consumption and stroke prevalence found in this study contradicts what other studies have shown [15, 16,23, 25, 64]. The findings from this study may be due to inappropriate measurement of alcohol consumption. Perhaps, inclusion of length, types and quantity of alcohol consumption in the analysis may have produced different results.

Further, the analysis shown here indicated that there was no statistically significant relationship between physical activity and stroke prevalence. In contrast, research has shown that the risk of stroke is lower for partially and fully active individuals compared to low active individuals [26]. An explanation for the finding from this study may be time-sequence based, or possibly a result of inadequate measurement. Therefore, in order to fully capture the true effects of the lifestyle factors on stroke prevalence, it will be important to consider re-conceptualizing these measures in the WHO SAGE survey. For instance, using the occupations of participants or other established measures of physical activity can be used to capture the association between physical activity and stroke prevalence. The associations between lifestyle factors and stroke prevalence observed in this study also indicate that perhaps there are other factors beyond the ‘traditional risk factors’ that are worth considering when looking at factors associated with stroke prevalence in Ghana.

### Comorbidities and stroke prevalence

This study showed that hypertension and diabetes were correlates of stroke. This is not surprising because studies have shown that hypertension and diabetes are the two leading risk factors of stroke [14, 17, 18]. This indicates the need for proper management of hypertension and diabetes in reducing the burden of stroke in Ghana. This study recommends that tackling hypertension and diabetes should be a major priority by the Ghana’s Ministry of Health. This may be achieved through routine screening of individuals in the country in order to enhance early detection of hypertension and diabetes in the population, before the age of 50.

### Limitations

This study has three key limitations. First, there may be possible under-estimation of stroke prevalence in this study. Measurement of stroke prevalence was based on self-report and it is likely that some of the respondents did not report living with a stroke most especially if this was not visible to the research staff. Second, some of the findings may be time-sequencing challenged with respect to determining the factors associated with stroke. For instance, it is possible that some of the socioeconomic and demographic characteristics of the stroke survivors may have changed after diagnosis. Hence, these characteristics may not determine stroke prevalence but may rather be consequences of stroke. For instance, it is possible that those who have been diagnosed of stroke stopped working and that was why those who were unemployed had higher odds of stroke. It would have been good to know the employment status of the stroke survivors before and after the stroke diagnosis; this would have revealed the true effect of employment status on stroke prevalence. Nonetheless, the findings from this study suggested that living with stroke affect the employment status of an individual and this is worth paying attention to.

Further, the findings suffered from the problematic nature of measurements for some of the lifestyle factors. For instance, the measure of physical activity in this study was based on the number of days respondents spent doing recreational activities such as sports, fitness, or recreational leisure activities. Based on this measure, 86.5% of Ghanaian adults were not physically active. This may be erroneous because recreational activities were used as proxies for physical activity and this may have led to under-estimating the physically active in this study. In addition, using recreational leisure as a proxy for physical activity also ignores other forms of physical activity such as that associated with blue collar work and street training; this may have biased some of our estimates. We recommend that the findings should be interpreted with caution.

Lastly, the measures for smoking and alcohol consumption may have contributed to the non-statistically significant associations between smoking, alcohol consumption and stroke prevalence. The questions on smoking and alcohol consumption focused on whether an individual had ever smoked/consumed alcohol and/or currently smoking/consuming alcohol. However, there were no question on the frequency of smoking/consumption of alcohol or types of alcohol consumed; these may be better measures of smoking or alcohol consumption to capture the effect of smoking/alcohol on stroke prevalence.

Despite these limitations, this is the first study in Ghana that used nationally representative sample to examine the prevalence and correlates of stroke. As a result, the findings from this study can be generalized to Ghanaians who are 50 years and above.

## Conclusion

This study examined the prevalence and correlates of stroke in Ghana, among people aged 50 years and above. The results suggest that interventions to reduce stroke prevalence and impact must be developed alongside interventions for hypertension, diabetes and sociodemographic/economic factors such as marital status, level of education, and employment status. The findings also raised concerns about re-conceptualizing the measures of lifestyle factors (i.e. smoking, alcohol consumption and physical activity) used in the WHO SAGE Survey in a way that reflects the Ghanaian setting. Finally, this study suggests that examining the correlates of stroke should go beyond the traditional risk factors to exploring other plausible non-traditional risk factors that may be associated with stroke.

## Supporting information

S1 Table Multicollinearity tests. (DOCX)

## References

[1] Feigin VL, Forouzanfar MH, Krishnamurthi R, Mensah GA, Connor M, Bennett DA, et al Global Burden of Diseases, Injuries, and Risk Factors Study 2010 (GBD 2010) and the GBD Stroke Experts Group. Global and regional burden of stroke during 1990–2010: findings from the global burden of disease study 2010. Lancet. 2014;383(9913):245–54. 24449944 10.1016/s0140-6736(13)61953-4 PMC4181600

[2] World Heart Federation. The global burden of stroke World Health Organization, Geneva, Switzerland 2014 Available from: http://www.world-heart-federation.org/cardiovascular-health/stroke/. Cited 14 March 2015.

[3] United Nations. World population prospects: the 2015 revision. 2015. Available from: http://esa.un.org/unpd/wpp/publications/files/key_findings_wpp_2015.pdf. Cited 06 April 2015.

[4] World Health Organization. Towards a WHO long-term strategy for prevention and control of leading chronic diseases World Health Organization, Geneva, Switzerland 2004Available from: http://www.who.int/chp/knowledge/publications/en/LONG%20TERM%20STRATEGY%20Yach.pdf. Cited 14 March 2015.

[5] Agyemang C, Attah-Adjepong G, Owusu-Dabo E, de-Graft Aikins A, Addo J, Edusei AK, et al Stroke in Ashanti region of Ghana: an urgent need for action. Ghana Medical Journal. 2012;46(2):12–17. 23661812 PMC3645146

[6] Kengne AP, Anders CS. The neglected burden of stroke in sub-Saharan Africa. International Journal of Stroke. 2006;1:180–190. 10.1111/j.1747-4949.2006.00064.x 18706015

[7] Help Age International. Global AgeWatch Index 2015: Executive summary. 2015. Available from: https://www.helpage.org/global-agewatch/reports/global-agewatch-index-2015-insight-report-summary-and-methodology/#. Cited 02 February 2016.

[8] Sampane-Donkor ES, Owolabi MO, Bampoh P, Aspelund T, Gudnason V. Community awareness of stroke in Accra, Ghana. BMC Public Health. 2014;14:196 10.1186/1471-2458-14-196 24559414 PMC3943505

[9] Addo J, Agyemang C, Smeeth L, de-Graft Aikins A, Edusei AK, Ogedegbe O. A review of population-based studies on hypertension in Ghana. Ghana Medical Journal. 2012;46 (2):4–11. 23661811 PMC3645150

[10] Sanuade OA, Awuah RB, Kushitor M. Hypertension awareness, treatment and control in Ghana: a cross-sectional study. Ethnicity & Health. 2018 1–5. 10.1080/13557858.2018.1439898 29448808

[11] Sanuade OA, Boatemaa S, Kushitor MK. Hypertension prevalence, awareness, treatment and control in Ghanaian population: Evidence from the Ghana demographic and health survey. PLoS ONE. 2018;13(11):e0205985 10.1371/journal.pone.0205985 30403686 PMC6221286

[12] de-Graft Aikins A. Ghana's neglected chronic disease epidemic: a developmental challenge. Ghana Medical Journal. 2007;41:154–159. 18464903 PMC2350116

[13] Damasceno A, Gomes J, Azevedo A, Carrilho C, Lobo V, Lopes H. An epidemiological study of stroke hospitalizations in Maputo, Mozambique: A high burden of disease in a resource-poor country. Stroke. 2010;41:2463–2469. 10.1161/STROKEAHA.110.594275 20930157

[14] Lawes CM, Vander Hoorn S, Rodgers A. Global burden of blood-pressure-related disease, 2001. Lancet. 2008;371:1513–1518. 10.1016/S0140-6736(08)60655-8 18456100

[15] O'Donnell MJ, Xavier D, Liu L, Zhang H, Chin SL, Rao-Melacini P, et al Risk factors for ischaemic and intracerebral haemorrhagic stroke in 22 countries (the INTERSTROKE study): a case-control study. The Lancet. 2010;376(9735):112–23. 10.1016/S0140-6736(10)60834-3 20561675

[16] Owolabi MO, Sarfo F, Akinyemi R, Gebregziabher M, Akpa O, Akpalu A, et al Dominant modifiable risk factors for stroke in Ghana and Nigeria (SIREN): a case-control study. The Lancet Global Health. 2018; 6(4):e436–46. 10.1016/S2214-109X(18)30002-0 29496511 PMC5906101

[17] Eze CO, Agu CE, Kalu UA, Maduanusi CA, Nwali ST, Igwenyi C. The pattern and presentation of stroke in Federal Teaching Hospital Abakaliki (FETHA) south-east Nigeria. Journal of Biology, Agriculture and Healthcare. 2013;3(11):141–144.

[18] Toure K, Thiam A, Sene DF, Ndiave M, Coume M, Seck LB, et al Epidemiology of stroke at the Clinic of Neurology, Fann University Teaching Hospital, Dakar-Senegal. Dakar Medicine. 2008;53(2):105–110. 19634543

[19] Obiako OR, Oparah SK, Ogunniyi A. Prognosis and outcome of acute stroke in the University College Hospital Ibadan, Nigeria. Nigerian Journal of Clinical Practice. 2011;14(3):359–362. 10.4103/1119-3077.86784 22037085

[20] Wahab KW, Okubadejo NU, Ojini FI, Danesi MA. Predictors of short-term intra-hospital case fatality following first-ever ischemic stroke in Nigerians. Journal of The College of Physicians and Surgeons Pakistan. 2008;18(12):755–758. 19032888

[21] Nakayama T, Yokoyama T, Yoshiike N, Zaman MM, Date C, Tanaka H, et al Population attributable fraction of stroke incidence in middle-aged and elderly people: contributions of hypertension, smoking and atrial fibrillation. Neuroepidemiology. 2000;19:217–226. 10.1159/000026259 10859502

[22] Shah RS, Cole JW. Smoking and stroke: the more you smoke the more you stroke. Expert Review of Cardiovascular Therapy. 2010;8(7):917–32. 10.1586/erc.10.56 20602553 PMC2928253

[23] Emberson JR, Bennett DA. Effect of alcohol on risk of coronary heart disease and stroke; causality, bias, or a bit of both? Vascular Health and Risk Management. 2006;5(1):239–49. 10.2147/vhrm.2006.2.3.239 PMC1993990 17326330

[24] Kadlecová P, Andel R, Mikulík R, Handing EP, Pedersen NL. Alcohol consumption at midlife and risk of stroke during 43 years of follow-up cohort and twin analyses. Stroke. 2015;46(3):627–33. 10.1161/STROKEAHA.114.006724 25634001

[25] Hillbom M, Numminen H, Juvela S. Recent heavy drinking of alcohol and embolic stroke. Stroke. 1999;30(11):2307–12. 10548663 10.1161/01.str.30.11.2307

[26] Do Lee C, Folsom AR, Blair SN. Physical activity and stroke risk a meta-analysis. Stroke. 2003;34(10):2475–81. 10.1161/01.STR.0000091843.02517.9D 14500932

[27] Kurth T, Gaziano JM, Berger K, Kase CS, Rexrode KM, Cook NR, et al Body mass index and the risk of stroke in men. Arch Intern Med. 2002;162:2557–2562. 12456227 10.1001/archinte.162.22.2557

[28] Rost NS, Wolf PA, Kase CS, Kelly-Hayes M, Silbershatz H, Massaro JM, et al Plasma concentration of C-reactive protein and risk of ischemic stroke and transient ischemic attack: the Framingham study. Stroke. 2001;32(11):2575–9. 11692019 10.1161/hs1101.098151

[29] Sriram K, Benkovic SA, Miller DB, O’Callaghan JP. Obesity exacerbates chemically induced neurodegeneration. Neuroscience. 2002;115(4):1335–46. 12453501 10.1016/s0306-4522(02)00306-8

[30] Petrea RE, Beiser AS, Seshadri S, Kelly-Hayes M, Kase CS, Wolf PA. Gender differences in stroke incidence and poststroke disability in the Framingham Heart Study. Stroke. 2009;40(4):1032–7. 10.1161/STROKEAHA.108.542894 19211484 PMC2676725

[31] Danesi MA, Okubadejo NU, Ojini FI, Ojo OO. Incidence and 30-day case fatality rate of first-ever stroke in urban Nigeria: The prospective community-based epidemiology of stroke in Lagos (EPISIL) phase II results. Journal of the Neurological Sciences. 2013;331:43–47. 10.1016/j.jns.2013.04.026 23726277

[32] Connor MD, Walker R, Modi G, Warlow CP. Burden of stroke in black populations in sub-Saharan Africa. The Lancet Neurology. 2007;6(3):269–78. 10.1016/S1474-4422(07)70002-9 17303533

[33] American Heart Association. Heart disease and stroke statistics-at-a-glance. 2015. Available from: https://www.heart.org/idc/groups/ahamah-public/@wcm/@sop/@smd/documents/downloadable/ucm_470704.pdf. Cited 04 April 2016.

[34] Sanuade OA, Agyemang C. Stroke in Ghana: A systematic literature review In: de-Graft Aikins A, Agyei-Mensah S, Agyemang C, editors. Chronic Non-communicable diseases in Ghana: Multidisciplinary Perspectives. Sub-Saharan Publishers, Ghana; 2013 pp. 29–38.

[35] Avendano M, Kawachi I, Van Lenthe F, Boshuizen HC, Mackenbach JP, Van den Bos GA, et al Socioeconomic status and stroke incidence in the US elderly: The role of risk factors in the EPESE study. Stroke. 2006;37(6):1368–73. 10.1161/01.STR.0000221702.75002.66 16690902

[36] Kaplan GA, Keil JE. Socioeconomic factors and cardiovascular disease: a review of the literature. Circulation. 1993;88:1973–1998. 8403348 10.1161/01.cir.88.4.1973

[37] Xu F, Tse LA, Yin X, Yu IT, Griffiths S. Impact of socio-economic factors on stroke prevalence among urban and rural residents in Mainland China. BMC Public Health. 2008;8(1):1. 18495014 10.1186/1471-2458-8-170 PMC2416447

[38] Engels T, Baglione Q, Audibert M, Viallefont A, Mourji F, Faris MEA, et al Socioeconomic status and stroke prevalence in Morocco: results from the Rabat-Casablanca study. PLoS ONE. 2014;9(2):e89271 10.1371/journal.pone.0089271 24586649 PMC3938460

[39] Cox AM, McKevitt C, Rudd AG, Wolfe CDA. Socioeconomic status and stroke. Lancet Neurology. 2006;5:181–188. 10.1016/S1474-4422(06)70351-9 16426994

[40] World Health Organization. Social determinants of health World Health Organization, Geneva, Switzerland 2015 Available from: http://www.who.int/social_determinants/en/. Cited 12 December 2016.

[41] Ghana Statistical Service. 2010 Population and Housing Census: The elderly in Ghana. Accra;2013. Available from: https://www.ghanahealthservice.org/downloads/FINAL_23_6_%202010%20Facts%20and%20Figure%202010_30.10.14-1.pdf. Cited 14 January 2017.

[42] Ghana Statistical Service. Ghana Living Standard Survey Round 6 (GLSS) 6 Main Report. Accra; 2014.

[43] World Health Organization. Health statistics World Health Organization, Geneva, Switzerland;2016 Available from: http://www.who.int/countries/gha/en/

[44] Ghana Health Service. The health sector in Ghana: facts and figures 2010. Available from: http://www.moh-ghana.org/UploadFiles/Publications/GHS%20Facts%20and%20Figures%202010_22APR2012.pdf. Cited 14 January 2017.

[45] Medical and Dental Council, Ghana. Permanent register. Accra; 2018. Available from: http://mdcghana.org/gazette/. Cited 04 June 2018.

[46] Biritwum RB, Mensah G, Minicuci N, Yawson AE, Naidoo N, Chatterji S, et al Household characteristics for older adults and study background from SAGE Ghana Wave 1. Global Health Action. 2013;6. 10.3402/gha.v6i0.20096 PMC3681208 23759325

[47] Minicuci N, Biritwum RB, Mensah G, Yawson AE, Naidoo N, Chatterji S, et al Sociodemographic and socioeconomic patterns of chronic non-communicable disease among the older adult population in Ghana. Global Health Action. 2014;7. 10.3402/gha.v7.21292 PMC3991840 24746141

[48] Phaswana-Mafuya N, Peltzer K, Crampin A, Ahame E, Sokhela Z. Prevalence of self-reported diagnosed cataract and associated risk factors among elderly South Africans. International Journal of Environmental Research and Public Health. 2017;14(12):1523. 10.3390/ijerph14121523 PMC5750941 29211038

[49] Wu F, Guo Y, Kowal P, Jiang Y, Yu M, Li X, et al Prevalence of major chronic conditions among older Chinese adults: The Study on Global AGEing and adult health (SAGE) wave 1. PLoS ONE. 2013;8(9): e74176 10.1371/journal.pone.0074176 24069278 PMC3775793

[50] Venketasubramanian N, Tan LC, Sahadevan S, Chin JJ, Krishnamoorthy ES, Hong CY, et al Prevalence of stroke among Chinese, Malay, and Indian Singaporeans: a community-based tri-racial cross-sectional survey. Stroke. 2005;36(3):551–6. 10.1161/01.STR.0000155687.18818.13 15692124

[51] Jamrozik E, Hyde Z, Alfonso H, Flicker L, Almeida O, Yeap B, et al Validity of self-reported versus hospital-coded diagnosis of stroke: a cross-sectional and longitudinal study. Cerebrovascular Diseases. 2014;37(4):256–62. 10.1159/000358583 24686404

[52] Teh R, Doughty R, Connolly M, Broad J, Pillai A, Wilkinson T, et al Agreement between self-reports and medical records of cardiovascular disease in octogenarians. Journal of clinical epidemiology. 2013;66(10):1135–43. 10.1016/j.jclinepi.2013.05.001 23860185

[53] Engstad T, Bønaa KH, Viitanen M. Validity of self-reported stroke: the Tromsø Study. Stroke. 2000;31(7):1602–7. 10884460 10.1161/01.str.31.7.1602

[54] Bots ML, Looman SJ, Koudstaal PJ, Hofman A, Hoes AW, Grobbee DE. Prevalence of stroke in the general population: the Rotterdam Study. Stroke. 1996;27(9):1499–501. 8784119 10.1161/01.str.27.9.1499

[55] Bergmann MM, Byers T, Freedman DS, Mokdad A. Validity of self-reported diagnoses leading to hospitalization: a comparison of self-reports with hospital records in a prospective study of American adults. American Journal of Epidemiology. 1998;147(10):969–77. 9596475 10.1093/oxfordjournals.aje.a009387

[56] Okura Y, Urban LH, Mahoney DW, Jacobsen SJ, Rodeheffer RJ. Agreement between self-report questionnaires and medical record data was substantial for diabetes, hypertension, myocardial infarction and stroke but not for heart failure. Journal of Clinical Epidemiology. 2004;57(10):1096–103. 10.1016/j.jclinepi.2004.04.005 15528061

[57] Jin YP, Di Legge S, Østbye T, Feightner JW, Saposnik G, Hachinski V. Is stroke history reliably reported by elderly with cognitive impairment? A community-based study. Neuroepidemiology. 2010;35(3):215–20. 10.1159/000315484 20664296

[58] WHO. Study of Global Ageing and Adult Health- Survey manual. 2006. Available from: http://www.who.int/healthinfo/survey/SAGESurveyManualFinal.pdf. Cited 08 January 2019.

[59] Wong CW, Kwok CS, Narain A, Gulati M, Mihalidou AS, Wu P, et al Marital status and risk of cardiovascular diseases: a systematic review and meta-analysis. Heart. 2018;104:1937–1948. 10.1136/heartjnl-2018-313005 29921571

[60] Sanuade OA. Burden of stroke in Ghana: Prevalence, experience and caregiving. Doctoral Thesis, University of Ghana. 2016. Available from: http://ugspace.ug.edu.gh/handle/123456789/22927

[61] Lai SM, Studenski S, Duncan PW, Perera S. Persisting consequences of stroke measured by the Stroke Impact Scale. Stroke. 2002;33(7):1840–1844. 12105363 10.1161/01.str.0000019289.15440.f2

[62] Birabi BN, Oke KI, Dienve PO, Okafor UC. Cost burden of post stroke condition in Nigeria: a pilot study. Global Journal of Health Sciences. 2012;4(6):17–22. 10.5539/gjhs.v4n6p17 PMC4776983 23121738

[63] Owusu-Dabo E, Lewis S, McNeill A, Gilmore A, Britton J. Smoking uptake and prevalence in Ghana. Tobacco control. 2009;18(5):365–70. 10.1136/tc.2009.030635 19581276 PMC2745559

[64] Gorelick PB, Rodin MB, Langenberg P, Hier DB, Costigan J. Weekly alcohol consumption, cigarette smoking, and the risk of ischemic stroke Results of a case‐control study at three urban medical centers in Chicago, Illinois. Neurology. 1989;39(3):339 2927640 10.1212/wnl.39.3.339

