# Supplementary material for: Prevalence and correlates of stroke among older adults in Ghana: Evidence from the Study on Global AGEing and adult health (SAGE)
Source: PLoS One. 2019 Mar 13;14(3):e0212623. doi: 10.1371/journal.pone.0212623 (PMC6415815; doi:10.1371/journal.pone.0212623)
Supplement: S1 Table — (DOCX) [file pone.0212623.s001.docx]

**S1 Table. Multicollinearity tests.**

| **Variable** | **VIF** | **1/VIF** |
| --- | --- | --- |
| **Sex** |  |  |
| Male | 1.94 | 0.51633 |
| Female | - | - |
| **Age** |  |  |
| 50-59 | 3.99 | 0.25090 |
| 60-69 | 3.11 | 0.32159 |
| 70-79 | 2.63 | 0.38018 |
| 80+ | - | - |
| **Marital status** | |  |
| Currently married | - | - |
| Never married | 1.03 | 0.97238 |
| Separated/divorced | 1.34 | 0.74733 |
| Widowed | 1.6 | 0.54634 |
| **Place of residence** | |  |
| Rural |  |  |
| Urban | 1.37 | 0.72935 |
| **Level of education** | |  |
| No education | - | - |
| Primary | 1.34 | 0.74835 |
| Secondary | 1.18 | 0.84741 |
| Higher | 1.60 | 0.62371 |
| **Wealth status** | |  |
| Poorest | 2.23 | 0.44777 |
| Poorer | 2.02 | 0.49529 |
| Middle | 1.86 | 0.53702 |
| Richer | 1.75 | 0.5709 |
| Richest | - | - |
| **Employment status** | | |
| Unemployed | - | - |
| Employed | 1.22 | 0.81668 |
| **Ethnicity** | |  |
| Akan | - | - |
| Ewe | 1.10 | 0.90517 |
| Ga- Adangbe | 1.18 | 0.84916 |
| Gruma | 1.16 | 0.86230 |
| Guan | 1.04 | 0.96527 |
| Mande Busanga | 1.05 | 0.95537 |
| Mole Dagbani | 1.25 | 0.80143 |
| Others | 2.05 | 0.48771 |
| **Smoking Status** | |  |
| Non-smoker | - | - |
| Current smokers | 1.30 | 0.77175 |
| Previous smokers | 1.18 | 0.84802 |
| **Alcohol consumption status** | | |
| Non-drinkers | - | - |
| Occasional drinkers | 6.89 | 0.14515 |
| Regular drinkers | 7.02 | 0.14252 |
| **Physical Activity** | |  |
| Fully active | - | - |
| Partially active | 1.59 | 0.6309 |
| Inactive | 1.65 | 0.60723 |
| **BMI Status** | |  |
| Normal | - | - |
| Underweight | 1.19 | 0.83955 |
| Overweight | 1.20 | 0.83635 |
| Obese | 1.30 | 0.76935 |
| **Hypertension** | |  |
| No | - | - |
| Yes | 1.17 | 0.85457 |
| **Diabetes** |  |  |
| No | - | - |
| Yes | 1.06 | 0.94485 |
